# Laser Devices and Autologous Platelet Concentrates in Prevention and Treatment of Medication-Related Osteonecrosis of the Jaws: A Systematic Review

**DOI:** 10.3390/medicina59050972

**Published:** 2023-05-18

**Authors:** Andrea Scribante, Martina Ghizzoni, Matteo Pellegrini, Federica Pulicari, Francesco Spadari

**Affiliations:** 1 Section of Dentistry, Department of Clinical, Surgical, Diagnostic and Pediatric Sciences, University of Pavia, 27100 Pavia, Italy; martina.ghizzoni01@universitadipavia.it; 2 Maxillo-Facial Surgery and Dental Unit, Fondazione IRCCS Cà Granda Ospedale Maggiore Policlinico, 20122 Milan, Italy; matteo.pellegrini@unimi.it (M.P.); federica.pulicari@studenti.unimi.it (F.P.); francesco.spadari@unimi.it (F.S.); 3 Department of Biomedical, Surgical and Dental Sciences, University of Milan, Via della Commenda 10, 20122 Milan, Italy

**Keywords:** autologous-platelet concentrates, bisphosphonates, dentistry, low-level-laser therapy, medication-related osteonecrosis of the jaw, MRONJ prevention, MRONJ treatment, photobiomodulation, platelet-rich fibrin, platelet-rich plasma

## Abstract

*Background and Objectives:* Medication-related osteonecrosis of the jaws (MRONJ) is a disease that affects many patients taking anti-angiogenic and antiresorptive medicines. Since the pathogenetic mechanism is still partially unknown, preventive strategies, as well as treatment alternatives, are needed. Therefore, the aim of this research is to describe the main evidence from the last 10 years of clinical trials regarding the use of auxiliary devices such as autologous platelet concentrates (APCs) and laser, other than their effects against MRONJ disease onset or therapy. Advantages in the healing process and recurrence rates were also analyzed. *Materials and Methods:* A systematic search of the electronic databases of PubMed and Scopus was carried out. Data from the studies were analyzed, and the risk of bias was evaluated. *Results:* Nineteen studies between interventional studies, observational studies, and cohort studies have been considered in this review. *Conclusions:* Based on the studies included, the literature analysis shows that APCs could be a beneficial alternative in preventing and treating MRONJ. Laser technology, as a surgical tool or used on the antimicrobial photodynamic or photobiomodulation side, has been becoming increasingly popular in the last few years. The latest proposal concerning the combination of both auxiliary tools suggests interesting effects, but more studies should be conducted to evaluate eventual relapses and long-term consequences.

## 1. Introduction

Medication-related osteonecrosis of the jaw (MRONJ) is a condition associated with patients undergoing treatment with bisphosphonates or monoclonal antibodies for skeletal diseases, starting from milder conditions such as osteoporosis to more severe ones such as multiple myeloma or cancer metastases [1].

Marx was the first to describe this phenomenon in 2003, and today, it is recognized as a major side effect of these antiangiogenic and antiresorptive medications [2].

The American Association of Oral and Maxillofacial Surgeons (AAOMS), firstly in 2014, and then in an update in 2022, declared that MRONJ is determined by the presence of three criteria:

1. Bone exposure or bone detection by probing through a fistula in the oral cavity for at least 8 weeks.
2. Simultaneous or former therapy with antiresorptive or antiangiogenic drugs.
3. No history of radiotherapy or metastatic malignancies in the jaw area [3].

The literature suggests that the strategy for clinical management may vary according to the clinical examination, imaging, and the patient’s overall condition. However, its goal is to limit or even prevent patients’ pain, infection, and overall health depletion [4].

MRONJ can arise naturally or after invasive operations such as dental extractions. Either way, precautionary strategies and risk factor management are essential to circumventing jaws’ osteonecrosis [5,6]. 

The pathway including TGF-β1 could be involved in the occurrence of MRONJ disease, since it regulates bone matrix production and osteoblasts’ differentiation, mediating the bone remodeling process [7].

However, to date, what is known about the pathophysiology of MRONJ is based on animal studies, particularly in rats, and their consequent evaluation related to humans [8].

In the literature, different hypotheses have been reported, ranging from the inhibition of bone remodeling, immune system deficiency, soft tissue toxicity, and inflammatory or infectious mechanisms to the suppression of angiogenesis [9].

As mentioned above, due to the pathophysiologic mechanism remaining partially unknown and unsuccessful healing or frequent relapses, the research has recently focused more on improving standard procedures by adding supportive strategies. Auxiliary approaches include autologous platelet concentrates (APCs), hyperbaric oxygen, teriparatide, ozone therapy, and laser photobiomodulation therapy (PBMT) [10,11,12].

Autologous platelet concentrates are derived blood products; they were initially used in transfusional medicine to prevent and treat hemorrhages caused by severe platelet deficiency. They are higher than average in platelet concentration and are widely used in regenerative dentistry because of their capacity to improve wound healing and tissue regeneration [13].

Similarly, laser application promotes cell proliferation and differentiation. Additionally, it decreases pain and inflammation, leading to enhanced wound healing [14].

Currently, the gold standard for prevention and treatment techniques is still uncertain [15]. Therefore, this research aims to review the recent literature about MRONJ prevention and treatment proposals, analyzing adjunctive therapy options, specifically APCs and laser alone or in combination. 

## 2. Materials and Methods

### 2.1. Focused Questions

Are APCs and laser auxiliary devices useful to prevent and treat MRONJ disease? Are they able to enhance healing processes and reduce the recurrence rates of MRONJ disease?

### 2.2. Eligibility Criteria

The inclusion criteria [16] guiding this review were: (I) study model—interventional studies, observational studies, and cohort studies (II); participants—patients at high risk of MRONJ, patients with MRONJ and previous bisphosphonates treatment, and patients with recurrent MRONJ; (III) interventions—use of APCs and/or laser for MRONJ prevention and treatment; and (IV) outcome—the role of APCs and/or laser for MRONJ prevention or treatment, enhanced healing, and lower rates or no recurrence of MRONJ disease. Exclusively studies that adhere to all the inclusion criteria were examined. As regards the exclusion criteria, the following were considered: (I) abstracts of articles published in non-English languages; (II) duplicate studies; (III) irrelevant studies (full-text articles with purposes which were not appropriate to answer the question we focused on, contained the analysis of different supplementary treatments, or had full-text content not corresponding to the abstract); (IV) ex vivo or experimental animal studies; (V) studies with the absence of Ethics Committee approval; (VI) narrative reviews, systematic reviews, or systematic and meta-analysis reviews; (VII) case series and case reports. 

### 2.3. Search Strategy

The PICO model (Table 1) [17] (Population, Intervention, Comparison, Outcome) was used to conduct this review, through a literature search of the PubMed (MEDLINE) and Scopus electronic databases, based on the following three aspects: population (people at the high-risk stage or with MRONJ disease undergoing dental procedures), concept (evidence from clinical trials related to MRONJ prevention and MRONJ treatment and possible benefits from both strategies’ union), and context (in this regard, the review has not been circumscribed to any specific cultural element or setting).

Studies’ abstracts that analyzed the effects of platelet derivatives in the medication-related osteonecrosis of the jaws alone or related to laser treatment were reviewed.

During this scoping review of the literature, the preferred reporting items for scoping reviews (PRISMA) consensus was followed (Table S1 Supplementary Material) [18].

### 2.4. Research

The medical subject heading (MeSH) terms are bisphosphonates, Bisphosphonate-Related Osteonecrosis of the Jaw, Denosumab, Photobiomodulation Therapy, prevention, platelet-rich fibrin (PRF), platelet-rich plasma (PRP), treatment; an electronic search was performed in the PubMed (MEDLINE) and Scopus databases. Articles published in the years 2010 to 2022 were selected. The data extraction period was between November 2022 and March 2023. The last search was performed on 30 March 2023. Two calibrated reviewers (M.G. and M.P.) conducted the search. Disagreements and discrepancies were resolved by consensus, and three other reviewers were consulted (F.P., A.S., and F.S.). All the titles and abstracts were analyzed carefully from the articles searched first, and non-relevant studies were not included. All relevant articles were reviewed and scrutinized by analyzing full texts, documenting the findings, and recognizing any similar studies that matched the inclusion criteria selected. 

The present protocol has been registered within the Open Science Framework platform (Registration DOI-10.17605/OSF.IO/WFEP4).

The discussed strategies applied for each electronic database are exhibited in Table S2 (Supplementary Material).

### 2.5. Quality Assessment of Included Studies

This review was performed by evaluating the risk of bias by conducting a qualitative analysis of the clinical studies via the National Heart, Lung, and Blood Institute (NHLBI) Quality Assessment of Controlled Intervention Studies, for Observational Cohort and Cross-Sectional Studies [19].

## 3. Results

**Figure 1:**
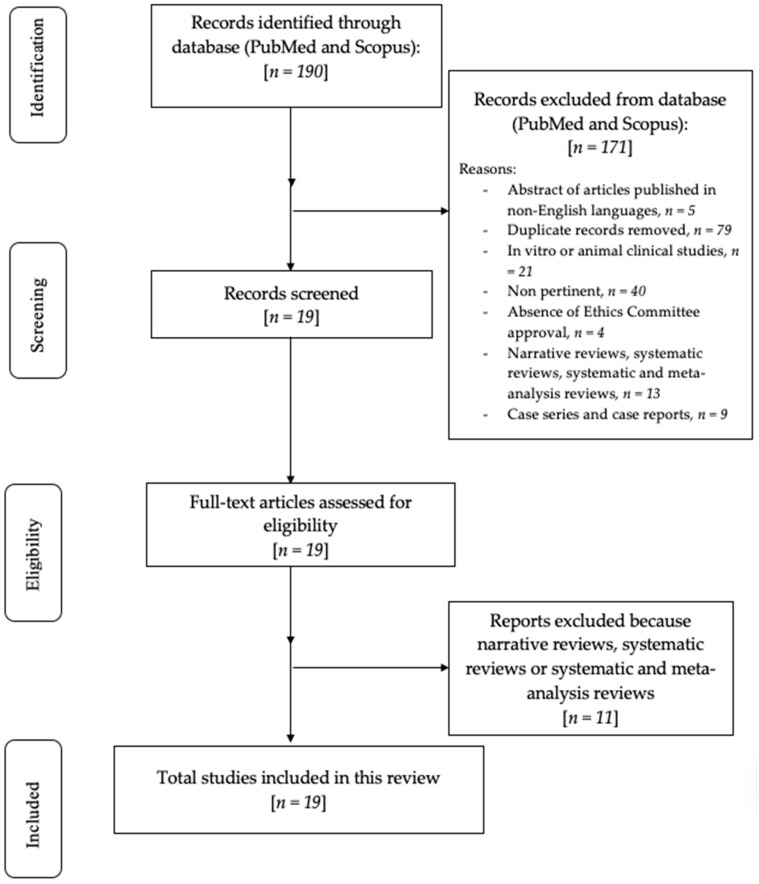
Flowchart of the review process.

The primary research detected 190 articles based on MeSH terms. After that, 171 articles were eliminated: 5 abstracts of articles published in non-English languages, 79 duplicates, 21 in vivo or experimental animal studies, 40 because they were irrelevant (not useful in answering the questions we focused on or had content not corresponding to the abstract), and 4 because of the absence of Ethics Committee approval. Moreover, 22 full-text articles were excluded, since they were narrative reviews, systematic reviews, and meta-analyses, alongside case series and case report studies. The 19 remaining articles assessed for eligibility were analyzed and finally included to be examined in this systematic review. The flowchart of the review procedure is described in Figure 1.

Table S3 (Supplementary Materials) presents the studies excluded from this systematic review and the motivations for their exclusion [20,21,22,23,24,25,26,27,28,29,30,31,32].

The studies included belonged to three different categories: controlled intervention studies [33,34,35,36,37,38,39,40], before–after (Pre–Post) studies with no control group [41,42], and observational cohort studies [43,44,45,46,47,48,49,50,51].

### Risk of Bias

The Cochrane Collaboration tool for assessing the risk of bias was adopted to evaluate the reviewed articles (Table 2). Table S4 (Supplementary Materials) shows the criteria for judging the risk of bias in the “risk of bias” assessment tool. This review shows a moderate risk of bias.

The baseline features of patients included in the examined studies are presented in Table 3.

Evidence of studies included in this systematic review (study design and aim, methods, results, and conclusions) is displayed in Table S5 (Supplementary Materials).

The NHLBI Quality Assessment Tool for Case-Control Intervention Studies is shown in Table S6 (Supplementary Materials). The NHLBI Quality Assessment Tool for Before-After (Pre-Post) Studies with No Control Group is shown in Table S7 (Supplementary Materials). The NHLBI Quality Assessment Tool for Observational Cohort and Cross-Sectional Studies is shown in Table S8 (Supplementary Materials). 

## 4. Discussion

Nineteen studies belonging to three different categories (controlled intervention studies, before–after (Pre–Post) studies with no control group, and observational cohort studies) were considered in this review. 

In terms of MRONJ, the main focus is to prevent its onset in high-risk patients and eventually treat the ones who have already developed it to minimize the impact on their well-being and slow the progression of their illness [52]. 

Therefore, this systematic review focuses on techniques to prevent MRONJ with autologous platelet concentrates and possibly laser surgery or aPDT and PBMT. It also aims to discuss the treatment of patients who have already developed osteonecrosis of the jaw (ONJ) by considering protocols involving APCs or laser alone or combined.

### 4.1. MRONJ Prevention Strategies 

To prevent MRONJ, knowing patients’ risk factors, such as pharmacological therapy including BPs, alveolar extraction, or senescence, and using prevention strategies are fundamental to reduce the rate of this complication since mucosal trauma can be a trigger for the disease’s onset [53].

The aim of these preventive interventions is to preserve or eventually re-establish oral health by managing hard- and soft-tissue-related risk factors. Among these local risk factors, it is possible to find oral infections such as periodontitis or peri-implantitis, anatomical features (torus, exostosis, or even pronounced mylohyoid ridge), and surgical procedures ranging from endodontic to regenerative [54]. 

However, a univocal protocol has not been identified yet. The literature shows a variety of different protocols, ranging from antibiotics to autologous platelet concentrates or even more innovative methods such as laser application [6].

In humans, PRP and PRF appeared to reduce MRONJ onset and promote early epithelization, speeding up recovery in patients treated with a bisphosphonate, because PRP is an autologous biomaterial abundant in growth factor, and PRF is a second-generation autologous product, whose role is crucial in controlling inflammation and accelerating immune response mediated by chemotactic molecules [33,34].

This suggests that PRF could interfere with bisphosphonate-induced effects regarding osteoclasts and mucosal cells [34].

PRGF belongs to the autologous product category that incorporates a copious amount of growth factors (TGF-beta, EGF, VEGF, IGF-1, BFGF, and HGF) that can be released simultaneously. Some of these factors, such as VEGF and PDGF, have the potential to trigger the mitosis of their target cells (endothelial and osteoblasts cells), leading to a positive effect in local administration [55].

Moreover, platelet-rich fibrin (PRF), because of its high representation of leukocytes contained inside the network of fibrin, has the role of defeating arising infection in those sites which have difficult healing processes [56]. 

For this reason, it should be considered as a dentist-friendly material in oral surgery in all cases where patients show a high risk of developing complications that can lead to infections such as osteomyelitis, to more severe scenarios such as osteonecrosis [36].

Patients with a high chance of developing MRONJ disease, who had a history of BPs and poor oral hygiene, treated with adjunctive therapy with L-PRF for tooth extraction showed no onset of MRONJ disease. Thus, it could be useful to decrease the rate of MRONJ in both oncologic and non-oncologic patients who need to undergo surgical procedures [35].

In patients with BP history, laser use after dentoalveolar surgery showed no signs of MRONJ after 6 months post-surgery [43].

As concerns laser application, aPDT and PBMT could be used as additional tools in post-exodontia healing since not even a single patient manifested signs or symptoms of MRONJ.

aPDT therapy does not have side effects and even gives bacterial resistance, in contrast to antibiotic treatment [43].

aPDT has been documented to have effects on several different types of cells, such as fibroblasts, osteoclasts, and keratinocytes, and even on angiogenesis, resulting in a boosted healing process [57].

On the other hand, PBMT targets one of the principal chromophores, namely cytochrome C oxidase, and up-regulates it. As a matter of fact, this leads to enhanced cell proliferation, migration, and differentiation, and thus an improved tissue healing process [58].

The combination of L-PRF and PBMT in patients cured with BPs showed physiological wound healing after a relatively short period of time of one month, and none of them experienced MRONJ [41].

### 4.2. Treatment Strategies

When osteonecrosis of the jaw is present, the first approach is usually noninvasive, based on antibiotics and antiseptics, but very often, a more invasive procedure is required [59].

This could lead to surgery to control the progression of bone loss; otherwise, the disease could proceed silently, invalidating patients’ quality of life [60].

The literature showed that the surgical removal of the necrotic bone leads to a better clinical outcome with complete soft tissue healing and a high rate of success [61].

Surgery carried out with an Er,Cr:YSGG laser combined with PRP is a successful strategy, as both are crucial in supplementing hard and soft tissues’ healing [47].

In addition, the effect that PRP might have on the regeneration of peripheral nerve fibers is currently being studied. In fact, some animal studies are confirming its effectiveness [62].

PDGF exhibits the chance to promote osteogenic progenitor differentiation and bone augmentation [63,64].

Among other APCs, L-PRF also seems to be a possible coupling treatment with surgical debridement in ONJ. Patients demonstrated the good maintenance of bone tissue and good overall healing [44,46].

PRF can effectively have beneficial effects in a short period, intended as a 1-month follow-up [37].

In all those cases where the wound closure for the first intention is not possible, PRF application can speed up the post-surgical re-epithelialization of the exposed site. It has been demonstrated that PRF application leads to a re-epithelization that occurs within two to four weeks [34,46].

In patients affected by ONJ, whose surgery was aided with PRP, results showed statistically significant success compared with the group who underwent surgery alone [51].

As concerns L-PRF applications, recent research has shown that the mixture of L-PRF with (bone morphogenetic protein-2) BMP-2 might have favorable outcomes. It could have a key role in contrasting the inhibition of bone remodeling processes that underlie MRONJ by intensifying them [40].

Regarding PBMT in association with surgery and PRF and its alternative forms (L-PRF and A-PRF), recent research in humans indicates that correct bone healing and regeneration is performed [38,45].

PBMT is revealed to be useful for angiogenesis, calcium deposition, and osteogenic cell proliferation as well as tissue healing [65].

Moreover, it has been demonstrated that PBMT inhibits keratinocytes apoptosis induced by alendronate, alongside other well-known effects such as cell migration, proliferation, and differentiation. Thus, PMBT, by favoring the migration of keratinocytes, other than angiogenesis, contributes to oral tissue wound healing [66].

The combination of antiseptic effects of antibiotics, L-PRF, and its proven consequences on wound healing and PBMT seemed to be an effective option for ONJ treatment [38].

Research showed that in patients with a medical background of MRONJ, dental extractions followed by PMBT can effectively prevent the relapsing of the disease. Patients healed their mucosa after 2 weeks, comparable to healthy patients, without any complications or prolonged treatment time [39].

For stage zero (prodromal disease) and stage I, intended as bone exposure without any symptoms and no infection, the approach involves antibiotic therapy alone or combined with chlorhexidine washes. As AAOMS suggests, analgesics can be administered as conservative therapy in the case of asymptomatic MRONJ lesions. However, they can also be prescribed for 3–5 days after surgery [35,40,44].

This conservative approach suggested by AAOMS, in some critical cases, can be strengthened with adequate surgical approaches involving superficial surgical debridement [3,62].

As the MRONJ lesion grades increase, the AAOMS guidelines indicate a more structured treatment [3]. 

As regards stage II, the AAOMS guidelines suggest counteracting inflammation and infection with soft tissue debridement combined with necrotic bone asportation. In stage III, the approach contemplates the surgical resection of the necrotic site [67]. 

In these cases, patients undergo general anesthesia and marginal bone resection, typically involving the entire alveolar process, till vital, bloody borders [62].

However, traditional surgical approaches lead to several drawbacks including: extended periods of hospitalization, convalescence, decreases in quality of life for the patients; increased risk rates of relapses with a chance of augmented areas of necrosis, and the consequent need for a second surgery; infections or bone fractures; complications that lead to the interruption of chemotherapy, or in the worst-case scenario, final treatment failure [68]. 

Grade II MRONJ should be treated with PMBT which is demonstrated to have several benefits such as the regulation of osteoblasts’ metabolism, proliferation, and differentiation; speeding up wound healing; and reducing discomfort and pain [49].

For grades II and III, the combination of piezo surgery, APCs, and even Nd: YAG bio-stimulation leads to a promising management approach [48].

The synergy of two different lasers, the Er: YAG laser for osteonecrosis removal and the diode laser for PBMT, associated with PRP, seemed to improve tissue healing, acting on keratinocytes, endothelial cells, and osteoblasts, as well as eliminating the need for painkillers [42].

The combined effect of medical, laser-assisted surgery, and PMBT thus seems to be a successful approach [49].

Finally, it should be considered that other uninvestigated aspects could significantly affect the oral environment. Endogenous commensal microbiota could play a crucial role in the severity of ONJ in high-risk patients [69]; thus, microbial dysbiosis needs to be kept under control [70]. Compounds such as bacterial lysates [71] and other natural composites [72] can change clinical and microbiological individual variables. Thereby, they could have a role in MRONJ prevention.

Additionally, other treatments such as the use of ozone [73] and other hydrogels [74] showed promising results. Variables should be examined in future clinical trials. 

However, this report has some limitations. The electronic research did not include any type of information specialists or academic librarians. Perhaps the search procedure could have been too specific for a scoping question. The inclusion of both at-risk MRONJ patients and patients with MRONJ introduced a lot of heterogeneity into the study, as well. Moreover, it was difficult to compare results that might have varied depending on the sample considered; indeed, the autologous platelet concentrates may have differed considering individual variables. The same applies for lasers that can change in features and parameters based on the producer company. The high rate of clinical improvement or healing in the use of modern measures such as APCs or lasers is only measured qualitatively and not quantitatively. The heterogeneity of materials available on the market and the potentially infinite combinations of options make the correlation among them a complex topic. 

Future studies are needed to study long-term outcomes concerning APCs’ applications alone or in combination with lasers both in preventing MRONJ’s occurrence and its treatment. Moreover, combining both treatment strategies (APCs + PBMT) should be further investigated with larger sample sizes and more randomized clinical trials. Eventually, a univocal protocol should be carried out on which professionals could rely so that MRONJ disease can be effectively dammed.

## 5. Conclusions

The literature analysis shows that autologous platelet concentrates (PRP; L-PRF; PRF; PDGF) could be beneficial auxiliary tools in the prevention and treatment of MRONJ disease. Additionally, laser technology, whether intended as a surgical device or used in the aPDT/PBMT mode, has been becoming increasingly utilized in clinical practice to counteract MRONJ in the last few years. Even the association of both APCs and laser PBMT promise great results but further studies, in particular randomized ones, and standardized protocols are needed.

## Figures and Tables

**Table 1:** This table outlines the PICO model followed.

1. Participants/population: patients at high-risk or with medication-related osteonecrosis of the jaw (MRONJ) disease
2. Intervention/exposure: autologous platelet concentrates (APCs) and/or laser treatment for MRONJ prevention and/or treatment.
3. Comparison/control: no comparison.
4. Outcomes: the role of APCs and/or laser for MRONJ prevention or treatment, enhanced healing, or lower rates or no recurrence of MRONJ disease.

**Table 2:** Risk of bias of the studies included in this review: the green symbol represents a low risk of bias, while the yellow symbol represents a high risk of bias.

	Random Sequence Generation	Allocation Concealment	Blinding	Incomplete Outcome Data	Selective Reporting
Mauceri et al., 2020 [33]	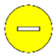	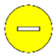	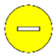	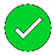	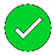
Asaka et al., 2017 [34]	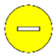	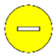	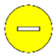	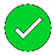	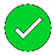
Parise et al., 2022 [35]	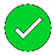	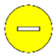	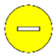	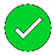	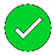
Miranda et al., 2021 [36]	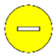	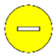	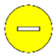	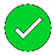	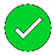
Giudice et al., 2018 [37]	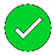	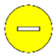	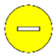	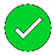	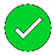
Tenore et al., 2020 [38]	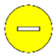	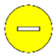	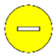	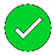	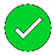
Vescovi et al., 2015 [39]	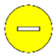	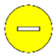	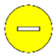	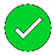	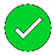
Park et al., 2017 [40]	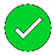	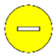	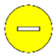	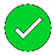	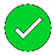
Sahin et al., 2020 [41]	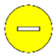	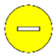	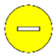	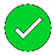	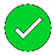
Merigo et al., 2018 [42]	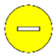	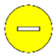	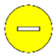	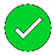	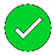
Tartaroti et al., 2020 [43]	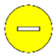	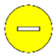	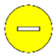	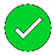	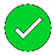
Ozalp et al., 2021 [44]	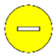	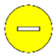	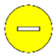	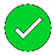	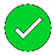
Martins et al., 2012 [45]	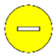	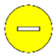	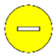	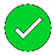	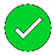
Valente et al., 2019 [46]	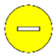	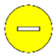	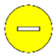	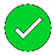	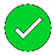
Mauceri et al., 2018 [47]	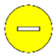	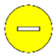	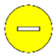	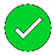	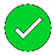
Sahin et al., 2021 [48]	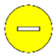	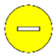	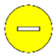	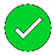	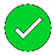
Vescovi et al., 2012 [49]	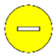	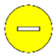	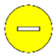	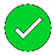	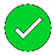
Nica et al., 2021 [50]	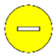	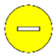	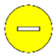	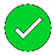	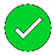
Longo et al., 2014 [51]	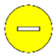	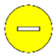	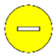	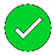	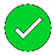

**Table 3:** Baseline characteristics of patients included in the selected studies.

Authors and Study Design	N° of Patients	% Women	Mean Age (Years), Mean (SD or Range)	Treatment Tested
Mauceri et al., 2020 Controlled intervention study [33]	Trial Group: 20 Control Group: 905	Trial Group: 80% Control Group: NR	Trial Group: 72.35 (±7.19) Control Group: NR	Platelet-rich plasma (PRP)
Asaka et al., 2017 Controlled intervention study [34]	Trial Group: 29 Control Group: 73	Trial Group: 89.6% Control Group: 91.7%	Trial Group: 73 (24–87) Control Group: 68 (33–88)	Platelet-rich fibrin (PRF)
Parise et al., 2022 Randomized controlled trial [35]	Group 1: 7 Group 2: 8 Group 3: 5	Group 1: 71.4% Group 2: 62.5% Group 3: 0.4%	Group 1: 59.42 (41–77) Group 2: 58.38 (41–73) Group 3: 71 (57–91)	L-PRF
Miranda et al., 2021 Retrospective controlled clinical trial [36]	Trial Group: 11 Control Group: 26	Trial group: 100% Control group: 96.15%	Trial group: 74.81 (SD: 8.88) Control group: 70.69 (SD: 8.03)	PRF
Giudice et al., 2018 Randomized controlled trial [37]	Trial Group: 24 Control Group: 23	Trial Group: 41.6% Control Group: 60.8%	Trial Group: 75.5 (±5.6) Control Group: 73.9 (±7.4)	PRF
Tenore et al., 2020 Retrospective controlled clinical study [38]	Trial Group: 13 Control Group: 8 Control Group:13	Trial Group: 61.5% Control Group: 100% Control Group: 76.9%	58.09 (45–92)	L-PRF + photobiomodulation therapy (PBMT)
Vescovi et al., 2015 Controlled clinical trial [39]	36	24/36 (66.67%)	68.5 (48–85)	Nd:YAG laser PBMT
Park et al., 2017 Randomized controlled trial [40]	Group L-PRF: 25 Group L-PRF + BMP-2: 30	Group L-PRF: 88% Group L-PRF + BMP-2: 96.7%	Group L-PRF: 75.24 (59–97) Group L-PRF + BMP-2: 75.2 (60–85)	PRF + bone morphogenetic protein-2 (BMP-2)
Sahin et al., 2020 Observational study [41]	44	32/44 (72.7%)	66.3	L-PRF + Nd:YAG laser PBMT
Merigo et al., 2018 Observational study [42]	21	16/21 (76.1%)	72.6 (60–85)	Er:YAG laser + PRP
Tartaroti et al., 2020 Prospective cohort study [43]	17	15/17 (88.2%)	73.37 (±9.97)	Antimicrobial photodynamic therapy (aPDT) + PBMT
Ozalp et al., 2021 Retrospective study [44]	13	7/13 (53.8%)	72.4 (54–84)	L-PRF
Martins et al., 2012 Retrospective study [45]	22	16/22 (72.7%)	58.1 (42–90)	PRP + PBMT
Valente et al., 2019 Retrospective study [46]	15	9/15 (60%)	64 (56–71)	L-PRF
Mauceri et al., 2018 Longitudinal cohort study [47]	10	7/10 (70%)	75.2 ± 5.94	Er,Cr:YSGG laser + PRP
Sahin et al., 2022 Retrospective cohort study [48]	21	14/21 (66.67%)	68.04 (49–85)	L-PRF + Nd:YAG laser
Vescovi et al., 2012 Retrospective study [49]	128	95/128 (74.2%)	NR	Antibiotic (G1) Antibiotic + LLLT (G2) Surgery (G3) Surgery + LLLT (G4)
Nica et al., 2021 Prospective observational study [50]	241	184/241 (76.34%)	67.7 (46–79)	PMBT (diode laser) + PRF
Longo et al., 2014 Retrospective observational study [51]	72	60/72 (83.3%)	59 (37–81)	PRP

## Data Availability

The data are available for use upon request to the corresponding author.

## Supplementary Materials

The following supporting information can be downloaded at: https://www.mdpi.com/article/10.3390/medicina59050972/s1, Table S1: PRISMA 2020 checklist; Table S2: Search strategies for electronic databases; Table S3: Summary table of studies excluded in this scoping review; Table S4: Criteria for judging risk of bias in the “Risk of bias” assessment tool; Table S5: Evidence of studies included in this scoping review; Table S6: NHLBI Quality Assessment Tool for Controlled Intervention Studies; Table S7: NHLBI Quality Assessment for Before-After (Pre-Post) Studies with No Control Group; Table S8: NHLBI Quality Assessment Tool for Observational Cohort and Cross-Sectional Studies.
