# Supplementary material for: Laser Devices and Autologous Platelet Concentrates in Prevention and Treatment of Medication-Related Osteonecrosis of the Jaws: A Systematic Review
Source: Medicina (Kaunas). 2023 May 18;59(5):972. doi: 10.3390/medicina59050972 (PMC10221057; doi:10.3390/medicina59050972)
Supplement: Supplementary file 1 [file medicina-59-00972-s001.zip › medicina-2387575-supplementary.pdf]

**Table S1.** PRISMA 2020 Checklist

| Section and Topic | Item # | Checklist item | Location where item is reported |
|---|---|---|---|
| **TITLE** | | | |
| Title | 1 | Identify the report as a systematic review. | Title |
| **ABSTRACT** | | | |
| Abstract | 2 | See the PRISMA 2020 for Abstracts checklist. | |
| **INTRODUCTION** | | | |
| Rationale | 3 | Describe the rationale for the review in the context of existing knowledge. | Initial introduction |
| Objectives | 4 | Provide an explicit statement of the objective(s) or question(s) the review addresses. | End of introduction |
| **METHODS** | | | |
| Eligibility criteria | 5 | Specify the inclusion and exclusion criteria for the review and how studies were grouped for the syntheses. | Dedicated section in M&M |
| Information sources | 6 | Specify all databases, registers, websites, organisations, reference lists and other sources searched or consulted to identify studies. Specify the date when each source was last searched or consulted. | Dedicated section in M&M |
| Search strategy | 7 | Present the full search strategies for all databases, registers and websites, including any filters and limits used. | Dedicated table |
| Selection process | 8 | Specify the methods used to decide whether a study met the inclusion criteria of the review, including how many reviewers screened each record and each report retrieved, whether they worked independently, and if applicable, details of automation tools used in the process. | Dedicated section in M&M |
| Data collection process | 9 | Specify the methods used to collect data from reports, including how many reviewers collected data from each report, whether they worked independently, any processes for obtaining or confirming data from study investigators, and if applicable, details of automation tools used in the process. | Dedicated section in M&M |
| Data items | 10a | List and define all outcomes for which data were sought. Specify whether all results that were compatible with each outcome domain in each study were sought (e.g. for all measures, time points, analyses), and if not, the methods used to decide which results to collect. | Dedicated section in M&M |
| | 10b | List and define all other variables for which data were sought (e.g. participant and intervention characteristics, funding sources). Describe any assumptions made about any missing or unclear information. | Dedicated section in M&M |
| Study risk of bias assessment | 11 | Specify the methods used to assess risk of bias in the included studies, including details of the tool(s) used, how many reviewers assessed each study and whether they worked independently, and if applicable, details of automation tools used in the process. | 3 reviewers assessed the risk of bias – specified in M&M |
| Effect measures | 12 | Specify for each outcome the effect measure(s) (e.g. risk ratio, mean difference) used in the synthesis or presentation of results. | Mean difference (M&M) |
| Synthesis methods | 13a | Describe the processes used to decide which studies were eligible for each synthesis (e.g. tabulating the study intervention characteristics and comparing against the planned groups for each synthesis (item #5)). | Type of intervention |
| | 13b | Describe any methods required to prepare the data for presentation or synthesis, such as handling of missing summary statistics, or data | Procedure described |

| Section and Topic | Item # | Checklist item | Location where item is reported |
|---|---|---|---|
| | | conversions. | M&M |
| | 13c | Describe any methods used to tabulate or visually display results of individual studies and syntheses. | Procedure described M&M |
| | 13d | Describe any methods used to synthesize results and provide a rationale for the choice(s). If meta-analysis was performed, describe the model(s), method(s) to identify the presence and extent of statistical heterogeneity, and software package(s) used. | Answer to PICO |
| | 13e | Describe any methods used to explore possible causes of heterogeneity among study results (e.g. subgroup analysis, meta-regression). | N/A |
| | 13f | Describe any sensitivity analyses conducted to assess robustness of the synthesized results. | N/A |
| Reporting bias assessment | 14 | Describe any methods used to assess risk of bias due to missing results in a synthesis (arising from reporting biases). | N/A |
| Certainty assessment | 15 | Describe any methods used to assess certainty (or confidence) in the body of evidence for an outcome. | Reported conclusions of the included studies |
| **RESULTS** | | | |
| Study selection | 16a | Describe the results of the search and selection process, from the number of records identified in the search to the number of studies included in the review, ideally using a flow diagram. | Dedicated table |
| | 16b | Cite studies that might appear to meet the inclusion criteria, but which were excluded, and explain why they were excluded. | Dedicated table |
| Study characteristics | 17 | Cite each included study and present its characteristics. | Dedicated table |
| Risk of bias in studies | 18 | Present assessments of risk of bias for each included study. | Dedicated table |
| Results of individual studies | 19 | For all outcomes, present, for each study: (a) summary statistics for each group (where appropriate) and (b) an effect estimate and its precision (e.g. confidence/credible interval), ideally using structured tables or plots. | Dedicated table |
| Results of syntheses | 20a | For each synthesis, briefly summarise the characteristics and risk of bias among contributing studies. | Dedicated table |
| | 20b | Present results of all statistical syntheses conducted. If meta-analysis was done, present for each the summary estimate and its precision (e.g. confidence/credible interval) and measures of statistical heterogeneity. If comparing groups, describe the direction of the effect. | N/A |
| | 20c | Present results of all investigations of possible causes of heterogeneity among study results. | Dedicated table |
| | 20d | Present results of all sensitivity analyses conducted to assess the robustness of the synthesized results. | Dedicated table |
| Reporting biases | 21 | Present assessments of risk of bias due to missing results (arising from reporting biases) for each synthesis assessed. | Dedicated table |
| Certainty of evidence | 22 | Present assessments of certainty (or confidence) in the body of evidence for each outcome assessed. | Dedicated table |
| **DISCUSSION** | | | |
| Discussion | 23a | Provide a general interpretation of the results in the context of other evidence. | Followed |

| Section and Topic | Item # | Checklist item | Location where item is reported |
|---|---|---|---|
| | 23b | Discuss any limitations of the evidence included in the review. | Followed |
| | 23c | Discuss any limitations of the review processes used. | Followed |
| | 23d | Discuss implications of the results for practice, policy, and future research. | Followed |
| **OTHER INFORMATION** | | | |
| Registration and protocol | 24a | Provide registration information for the review, including register name and registration number, or state that the review was not registered. | DOI No 10.17605/OSF.IO/WFEP4 |
| | 24b | Indicate where the review protocol can be accessed, or state that a protocol was not prepared. | OSF |
| | 24c | Describe and explain any amendments to information provided at registration or in the protocol. | N/A |
| Support | 25 | Describe sources of financial or non-financial support for the review, and the role of the funders or sponsors in the review. | None |
| Competing interests | 26 | Declare any competing interests of review authors. | None |
| Availability of data, code and other materials | 27 | Report which of the following are publicly available and where they can be found: template data collection forms; data extracted from included studies; data used for all analyses; analytic code; any other materials used in the review. | N/A |

**Table S2:** Search strategies for electronic databases.

| Database | Search strategy |
|---|---|
| PubMed (MEDLINE) | #1 "PRF" [MESH] OR (Fibrin, Platelet-Rich) OR (Platelet Rich Fibrin) OR (L-PRF) OR (Leukocyte- and Platelet-Rich Fibrin) OR (Leukocyte and Platelet Rich Fibrin)<br><br>#2 "Low-Level Light Therapy" [MESH] OR (Light Therapy, Low-Level) OR (Low Level Light Therapy) OR (Low-Level Light Therapies) OR (Photobiomodulation Therapy) OR (LLLT) OR (Low Level Laser Therapy) OR (Low-Power Laser Irradiation) OR (Laser Biostimulation) OR (Laser Phototherapy)<br><br>#3 "Disphosphonates" [MESH] OR (Bisphosphonates) OR (Bisphosphonate)<br><br>#4"Denosumab" [MESH] (Xgeva) or (AMG 162) or (Prolia)<br><br>#5"Bisphosphonate-Associated Osteonecrosis of the Jaw" [MESH] OR (Bisphosphonate-Induced Osteonecrosis of the Jaw) OR (Bisphosphonate-Related Osteonecrosis of the Jaw) OR (Bisphosphonate-Associated Osteonecrosis) OR (Bisphosphonate Osteonecrosis)<br><br>#6 "Platelet-Rich Plasma" [MESH] OR (Plasma, Platelet-Rich) OR (Platelet Rich Plasma)<br><br>#7 "Prevention and control [Subheading]" [MESH] OR (prophylaxis) OR (preventive therapy) OR (prevention and control) OR (preventive measures) OR (prevention) OR (control)<br><br>#8 "Therapy [Subheading]" [MESH] OR (treatment) OR (disease management)<br><br>#9 #1 OR #2 OR #3 OR #4 AND #5<br><br>#10 #1 AND #3 AND #7 OR #8<br><br>#11 #2 AND #5 AND #7 OR #8<br><br>#12 #4 AND #5 AND #7 OR #8 |

| | |
|---|---|
| SCOPUS | #1 “PRF” [MESH] OR (Fibrin, Platelet-Rich) OR (Platelet Rich Fibrin) OR (L-PRF) OR (Leukocyte- and Platelet-Rich Fibrin) OR (Leukocyte and Platelet Rich Fibrin)<br><br>#2 “Low-Level Light Therapy” [MESH] OR (Light Therapy, Low-Level) OR (Low Level Light Therapy) OR (Low-Level Light Therapies) OR (Photobiomodulation Therapy) OR (LLLT) OR (Low Level Laser Therapy) OR (Low-Power Laser Irradiation) OR (Laser Biostimulation) OR (Laser Phototherapy)<br><br>#3 “Disphosphonates” [MESH] OR (Bisphosphonates) OR (Bisphosphonate)<br><br>#4“Denosumab” [MESH] (Xgeva) or (AMG 162) or (Prolia)<br><br>#5“Bisphosphonate-Associated Osteonecrosis of the Jaw” [MESH] OR (Bisphosphonate-Induced Osteonecrosis of the Jaw) OR (Bisphosphonate-Related Osteonecrosis of the Jaw) OR (Bisphosphonate-Associated Osteonecrosis) OR (Bisphosphonate Osteonecrosis)<br><br>#6 “Platelet-Rich Plasma” [MESH] OR (Plasma, Platelet-Rich) OR (Platelet Rich Plasma)<br><br>#7 “Prevention and control [Subheading]” [MESH] OR (prophylaxis) OR (preventive therapy) OR (prevention and control) OR (preventive measures) OR (prevention) OR (control)<br><br>#8 “Therapy [Subheading]” [MESH] OR (treatment) OR (disease management)<br><br>#9 #1 OR #2 OR #3 OR #4 AND #5<br><br>#10 #1 AND #3 AND #7 OR #8<br><br>#11 #2 AND #5 AND #7 OR #8<br><br>#12 #4 AND #5 AND #7 OR #8 |

**Table S3.** Summary table of studies excluded in this systematic review.

| Excluded Studies | Exclusion Reasons |
|---|---|
| Reis et al., 2022 [1] | Systematic Review |
| Razavi et al., 2022 [2] | Systematic Review |
| Cano-Duràn et al., 2017 [3] | Narrative review |
| De Santis et al., 2020 [4] | Narrative review |
| Mijiritsky et al., 2022 [5] | Narrative review |
| Rusilas et al., 2020 [6] | Narrative review |
| Lopez-Jornet et al., 2016 [7] | Systematic Review |
| Fortunato et al., 2020 [8] | Systematic Review |
| Hao et al., 2022 [9] | Narrative review |
| De Souza Tolentino et al., 2019 [10] | Systematic Review |
| Goker et al., 2021 [11] | Systematic Review |
| Momesso et al., 2020 [12] | Meta-analysis |
| Li et al., 2020 [13] | Systematic Review |

**Table S4.** Criteria for judging risk of bias in the "Risk of bias" assessment tool.

| | |
|---|---|
| **Random Sequence Generation** | |
| Criteria for a judgement of 'Low risk' of bias. | The investigators describe a random component in the sequence generation process. |
| Criteria for the judgement of 'High risk' of bias. | The investigators describe a non-random component in the sequence generation process. Usually, the description would involve some systematic, non-random approach.<br>Other non-random approaches happen much less frequently than the systematic approaches mentioned above and tend to be obvious. They usually involve judgement or some method of non-random categorization of participants. |
| **Allocation Concealment** | |
| Criteria for a judgement of 'Low risk' of bias. | Participants and investigators enrolling participants could not foresee assignment because one of the following, or an equivalent method, was used to conceal allocation. |
| Criteria for the judgement of 'High risk' of bias. | Participants or investigators enrolling participants could possibly foresee assignments and thus introduce selection bias. |
| **Blinding** | |
| Criteria for a judgement of 'Low risk' of bias. | Any one of the following:<br>- No blinding or incomplete blinding, but the review authors judge that the outcome is not likely to be influenced by lack of blinding;<br>- Blinding of participants and key study personnel ensured, and unlikely that the blinding could have been broken;<br>- No blinding of outcome assessment, but the review authors judge that the outcome measurement is not likely to be influenced by lack of blinding;<br>- Blinding of outcome assessment ensured, and unlikely that the blinding could have been broken. |
| Criteria for the judgement of 'High risk' of bias. | Any one of the following:<br>- No blinding or incomplete blinding, and the outcome is likely to be influenced by lack of blinding;<br>- Blinding of key study participants and personnel attempted, but likely that the blinding could have been broken, and the outcome is likely to be influenced by lack of blinding; |

| | |
|---|---|
| | - No blinding of outcome assessment, and the outcome measurement is likely to be influenced by lack of blinding;<br>- Blinding of outcome assessment, but likely that the blinding could have been broken, and the outcome measurement is likely to be influenced by lack of blinding. |
| **Incomplete Outcome Data** | |
| Criteria for a judgement of 'Low risk' of bias. | Any one of the following:<br>- No missing outcome data;<br>- Reasons for missing outcome data unlikely to be related to true outcome (for survival data, censoring unlikely to be introducing bias);<br>- Missing outcome data balanced in numbers across intervention groups, with similar reasons for missing data across groups;<br>- For dichotomous outcome data, the proportion of missing outcomes compared with observed event risk not enough to have a clinically relevant impact on the intervention effect estimate;<br>- For continuous outcome data, plausible effect size (difference in means or standardized difference in means) among missing outcomes not enough to have a clinically relevant impact on observed effect size;<br>- Missing data have been imputed using appropriate methods. |
| Criteria for the judgement of 'High risk' of bias. | Any one of the following:<br>- Reason for missing outcome data likely to be related to true outcome, with either imbalance in numbers or reasons for missing data across intervention groups;<br>- For dichotomous outcome data, the proportion of missing outcomes compared with observed event risk enough to induce clinically relevant bias in intervention effect estimate;<br>- For continuous outcome data, plausible effect size (difference in means or standardized difference in means) among missing outcomes enough to induce clinically relevant bias in observed effect size;<br>- 'As-treated' analysis done with substantial departure of the intervention received from that assigned at randomization;<br>- Potentially inappropriate application of simple imputation. |
| **Selective Reporting** | |

| | |
|---|---|
| Criteria for a judgement of 'Low risk' of bias. | Any one of the following:<br>- The study protocol is available and all of the study's pre-specified (primary and secondary) outcomes that are of interest in the review have been reported in the pre-specified way;<br>- The study protocol is not available but it is clear that the published reports include all expected outcomes, including those that were pre-specified (convincing text of this nature may be uncommon). |
| Criteria for the judgement of 'High risk' of bias. | Any one of the following:<br>- Not all of the study's pre-specified primary outcomes have been reported;<br>- One or more primary outcomes is reported using measurements, analysis methods or subsets of the data (e.g., subscales) that were not pre-specified;<br>- One or more reported primary outcomes were not pre-specified (unless clear justification for their reporting is provided, such as an unexpected adverse effect);<br>- One or more outcomes of interest in the review are reported incompletely so that they cannot be entered in a meta-analysis;<br>- The study report fails to include results for a key outcome that would be expected to have been reported for such a study. |

**Table S5.** Evidence of studies included in this systematic review.

| Authors and Year of Publication | Study Design and Aim | Methods | Results | Conclusions |
|---|---|---|---|---|
| Mauceri et al., 2020 [14] | A 2-year observational study, with 24 months follow-up, to compare standardized dental extraction procedures associated with (PRP), and conventional procedures in oncologic (ONC) and osteo-metabolic patients (OST) in danger of MRONJ. | 20 patients (6 ONC and 14 OST) treated with BFs underwent a standardized protocol for dental extraction combined with PRP. 63 tooth extraction were performed. As controls, historical cases, derived from the literature and including 171 ONC and 734 OST patients, were considered. | Success in PRP group. After two years from the surgery, no radiological or clinical signs of ONJ were found in any patient treated. No statistically significant differences were found when this datum was compared with historical controls ($P > 0.1$). | Including PRP in a standardized protocol could reduce the eventuality of MRONJ in both ONC and OST patients. |
| Asaka et al., 2017 [15] | A 3-months controlled trial study, to evaluate the effectiveness of platelet-rich fibrin (PRF) as a wound-healing accelerator in patients undergoing oral bisphosphonate therapy and requiring tooth extractions. | 102 patients were divided into a PRF group and a control group. | There were no intraoperative complications, and none of the patients exhibited onset of medication-related osteonecrosis of the jaw (MRONJ). | Early epithelization was confirmed in all PRF patients. Thus, PRF may reduce the risk of delayed recovery in patients undergoing oral bisphosphonate therapy. |
| Parise et al., 2022 [16] | A 6-months randomized controlled study, to evaluate the use of L-PRF in prevention and treatment of bone necrosis. | 20 patients, splitted in 3 groups (control; prevention and treatment). | The result of surgical treatment was successful for 57% in the control group, 100% in the prevention group, and 80% in the treatment group. | L-PRF allows the release of growth factors for a prolonged time, resulting in better healing, reducing the risk of contamination, edema, and postoperative pain, being a great ally in the prevention and treatment of MRONJ. |
| Miranda et al., 2021 [17] | A 6-months retrospective controlled clinical study to evaluate whether a PRF plug inserted in the post extraction socket can prevent the onset of MRONJ. | 37 patients in treatment with BFs, divided into two groups (control and study group), underwent 69 tooth extraction procedures. The study group received PRF post-extraction. | A slightly higher incidence of ONJ was found in treated patients in the control group. | The use of platelet concentrates in patients with high risk of MRONJ is a user-friendly technique with an excellent cost-benefit ratio in oral surgery. |

| | | | | |
|---|---|---|---|---|
| Giudice et al., 2018 [18] | A 1-year randomized monocentric controlled clinical study, to evaluate the efficacy of platelet-rich fibrin (PRF) after bone surgery compared to surgery alone in the treatment of medication-related osteonecrosis of the jaw (MRONJ). | 47 patients with diagnosis of stage II or III of MRONJ were recruited and divided in 2 groups (PRF and non-PRF group). Fisher's exact and Student t tests were used to evaluate differences between the 2 surgical protocols in terms of mucosal integrity, absence of infection, and pain evaluation at scheduled follow-ups of 1 month (T1), 6 months (T2), and 1 year (T3). | Analysis of mucosal integrity, absence of infection, and pain evaluation showed a significant difference between the 2 groups in favor of PRF only at T1, whereas no differences were determined at T2 and T3. | The results suggested that local application of PRF after bone surgery may improve the quality of life limited to the short-term follow-up and reduce pain and postoperative infections. |
| Tenore et al., 2020 [19] | A 6-months retrospective controlled clinical study, to compare the effect of three different treatment protocols on the healing outcome in patients with established medication-related osteonecrosis of the jaw (MRONJ). | 34 MRONJ patients were recruited from the Department database and were divided according to the treatment protocols in a study group (G1) and two control groups (G2 and G3). G1 was treated with antibiotic therapy, surgery, leukocyte- and platelet-rich fibrin (L-PRF), and PBMT; G2 was treated with antibiotic therapy and surgery; G3 was treated with antibiotic therapy and PBMT. | There was a significant association between the different treatment protocols and the outcomes at both three- and six-months follow-up. | The results show that the combination of antibiotic therapy, surgery, L-PRF, and PBMT may effectively contribute to MRONJ management. |
| Vescovi et al., 2015 [20] | A controlled clinical study, with mean follow-ups of 18.66 and 13.93 months, to validate the safety and efficacy of the protocol proposed in 2013, reporting the data related to its application in a particular category of patients under BPT at high risk for MRONJ and who were previously affected with MRONJ. | 36 patients previously affected with MRONJ, underwent 82 tooth extractions. Patients were divided in 2 groups: (G1) included extractions in patients treated before and healed from MRONJ in a different site from dental extraction procedure. (G2) included extractions in patients with MRONJ in the same site of dental extraction procedure. Mean follow-up was 18.66 months for G1 | In a total of 82 extractions, minimal bone exposure was observed in 2 cases, treated with Er:YAG Laser vaporization and then completely healed. | The data confirmed that Laser biostimulation is a reliable technique that can be considered in the surgical protocol for patients under BPT. |

| | | | | |
|---|---|---|---|---|
| | | and 13.93 months for G2 Antibiotic treatment was administered 3 days before and 2 weeks after tooth extractions. Patients were additionally treated with Nd:YAG PBMT, 5 applications of 1 minute each. Patients were evaluated 3 days and once a week for 2 months after the extractions and every time they received PMBT. | | |
| Park et al., 2017 [21] | A 16-weeks randomized prospective controlled study, to evaluate the effect of BMP-2 added to L-PRF on MRONJ. | 55 patients affected by MRONJ were divided into 2 groups (L-PRF +BMP-2 and L-PRF). Follow ups occurred 4 and 16 weeks post-surgery. | At follow-ups, patients in the L-PRF + BMP-2 showed favorable outcomes with complete resolution of the lesions, which was statistically significant compared with that of the therapy using L-PRF alone. | The combined use of BMP-2 and L-PRF leads to the early resolution of MRONJ; thus, patients who need to continue antiresorptive therapy may benefit from the combined regimen. |
| Sahin et al., 2020 [22] | An observational study, with a mean follow-up of 14.2 months, to investigate surgical methods applied in patients treated with bisphosphonates to prevent the onset of MRONJ after dentoalveolar surgery. | 44 patients in treatment with bisphosphonates, received a total of 63 surgeries. Carried out dentoalveolar procedure, antibiotics, put L-PRF in the socket and PBMT (Nd: YAG Laser). | Normal mucosal healing happened in one month, for all the patients without over time failures. | This procedure suggests an auspicious strategy to protect patients at risk of MRONJ, undergoing dentoalveolar surgery. |
| Merigo et al., 2018 [23] | An observational study, with a mean follow-up of 9.6 months, to present a combined approach of piezosurgery, PRP and Laser, for MRONJ management. | 21 patients, previously treated with antiresorptive or anti-angiogenic therapy, were diagnosed with MRONJ. They underwent combined treatment. Piezosurgery for removing the necrotic bone tissue and for obtaining the bone specimen | The post-operatory passed without discomfort and without the need of painkillers for all the patients. No bleeding and rapid healing process were shown. Almost all the | Sequential utilization of different high-technologies devices during all the steps of MRONJ treatment allows to perform a faster and less invasive surgery with a more comfortable postoperative healing process and it may represent a new and original |

| | | | | |
|---|---|---|---|---|
| | | essential for histological analysis; Er:YAG Laser to vaporize necrotic hard tissue until reaching the bleeding bone; PRP to stimulate tissues healing; and diode Laser (808 nm) as a biostimulant for the surgical site. | patients after at least 6 months follow-up, showed complete healing. | approach for treating this severe adverse event. |
| Tartaroti et al., 2020 [24] | A prospective cohort study, with a follow-up ranging from 3 to 29 months, and case series to investigate two different protocols in reference to photonics [antimicrobial photodynamic therapy (aPDT) and photobiomodulation (PBMT)] for prevention and treatment of MRONJ lesions | 18 patients were treated with preventive protocol by applying post-exo aPDT, followed by diode Laser irradiation, repeated weekly till complete tissue healing. | After a minimum of 6 months of follow up, no signs of MRONJ were noticed. | aPDT and PBMT therapy protocols seem to be effective as an adjuvant approach, in dentoalveolar extraction, for preventing MRONJ development. |
| Ozalp et al., 2021 [25] | A retrospective study, with a mean follow up of 20.1 ± 18.29 months, to evaluate the adjunctive role of L-PRF in surgically treated medication-related osteonecrosis of the jaws (MRONJ) patients. | 13 patients, nine of whom had stage III and four stage II MRONJ, underwent marginal resection, sequestrectomy with peripheral ostectomy (SPO) or curettage, and then L-PRF application. | All marginal resection and six SPO patients showed complete healing while four patients, who had SPO, or curettage experienced incomplete healing. | The use of L-PRF may be a favorable adjunctive option in the treatment of MRONJ owing to its favorable effects on tissue repair, ease of application, minimally invasive and cost-effective character, and autogenous nature. |
| Martins et al., 2012 [26] | A 6-months retrospective study, to analyze three different treatments on the healing results of bisphosphonate-related osteonecrosis of the jaws (BRONJ) in cancer patients. | 22 patients divided in 3 different treatment groups: pharmacological, surgical, and surgical + PRP and PBMT. | A significantly higher percentage of patients reached the current state of BRONJ without bone exposure in the PRP plus PMBT group than other groups after 1-month follow-up. | The combination of surgical approach + PRP and PBMT enhances BRONJ healing in oncologic patients. |
| Valente et al., 2019 [27] | A retrospective study, with a range of follow-up varying from 0.5 to 6 years, to analyze records from patients with documented | 15 patients, with a history of BPs or denosumab, underwent bone debridement/sequestrectomy + L-PRF and antibiotic therapy. | Treatment success set at 73.3%. PRF led to a total resolution in 11 of 14 cases in which it was used. | The use of PRF following bone debridement, showed potential benefits in the resolution of MRONJ. |

| | | | | |
|---|---|---|---|---|
| | medication-related osteonecrosis of the jaws. | | | |
| Mauceri et al., 2018 [28] | A 12-months longitudinal cohort study to evaluate the efficiency of a conservative surgical treatment combining Er,Cr:YSGG Laser and platelet-rich plasma (PRP) for the treatment of BRONJ in cancer patients. | 10 patients with MRONJ, after antibiotic therapy, were treated with Laser therapy combined with PRP. | Follow up at 12 months showed clinical improvement in 8 patients. | This study's findings suggest that a surgical approach combined with Er,Cr:YSGG Laser and PRP benefit cancer patients with general health issues. |
| Sahin et al., 2022 [29] | A retrospective cohort study, with a mean follow-up period of 18.04 ± 2.14 months, to evaluate the surgical technique described in the treatment of advanced stages of medication-related osteonecrosis of the jaw patients. | 21 patients affected by Stage 2-3 MRONJ were treated with ultrasonic piezoelectric bone surgery for necrotic bone removal, L-PRF and Nd:YAG Laser for biostimulation. Success was assessed as the maintenance of full mucosal coverage without signs of residual infection at 1 month 3, 6 and 12 months after surgery. | Complete mucosal healing was achieved in all patients at the third month. | The surgical protocol presented in this study shows promising results for surgical management of advanced stages of medication-related osteonecrosis of the jaw patients. |
| Vescovi et al., 2012 [30] | A retrospective analysis with long term follow-up (ranging from 6 to 54 months), to analyze the differences between surgical and conservative treatments, and also the efficacy of Er:YAG and Nd:YAG Lasers, in BRONJ disease. | 128 patients diagnosed with BRONJ had an overall of 151 sites of osteonecrosis, 101 of them treated. Sites were divided in 5 groups: G1(medical therapy); G2 (medical therapy +PMBT); G3 (medical + surgical therapy); G4 (medical therapy, surgical, including Laser-assisted therapy, and PMBT). Treatment results were evaluated referring to Ruggiero et al. system. | Clinical improvement was achieved in 25% BRONJ sites in G1, 66% in G2; 53% in G3 transitioned to a lower stage of BRONJ and 89% of the sites in G4 exhibit clinical amelioration. | The percentage of success obtained with a combined approach based on medical therapy, surgical (including Laser -assisted) therapy, and PMBT(G4) is significantly higher than the percentage of improvement obtained in G1, G2, and G3. |
| Nica et al., 2021 [31] | A prospective monocentric observational study, with a follow-up of at least 6 months, to design and apply a composed and stage- | 241 patients who were previously exposed to antiresorptive or anti-angiogenic therapy, as well as patients already diagnosed with | The healing proved to be complete, with spontaneous bone coverage in all the $n$ = 84 cases placed in an "at | Therefore, the clinical outcome of the present study indicates that patients with MRONJ in almost all stages of the disease can benefit from such a |

| | | | | |
|---|---|---|---|---|
| | approach therapy combining antibiotherapy, surgical treatment, and photo-biomodulation (PBMT) for the prevention or treatment of MRONJ lesions. | MRONJ at different stages of the disease were treated. A preventive protocol was applied for patients in an “at risk” stage. Patients staged “at-risk” for MRONJ and referred for tooth extraction received antibiotic treatment; dental extractions performed with minimum trauma. After curettage and lavage with saline solution, a suture was applied. In the next step, Diode Laser was used to photo-biomodulate the site, after tooth extraction, the PBM was performed at 24 h, 48 h, 72 h, day 4, day 5, day 6, and day 7, as well as 3 times/week for the following 2 weeks after surgery. The sutures were removed 10 days after the surgery. The patients in stage 0 of MRONJ received only antibiotic treatment and PBM, with no surgery. The treatment was received for 14 days. To reduce the local inflammation associated with pain, PBM was performed during the 7 consecutive days, followed by other 6 sessions of Laser irradiation distributed in the following 2 weeks. For all the patients in stages of MRONJ, the treatment protocol was antibiotic + preoperative PBM + surgery. To increase the healing by pre-conditioning the tissues, PBM was | risk" stage. For the $n = 49$ patients belonging to stage 0, pain reductions and decreases of mucosal inflammations were also obtained in all cases. For the $n = 108$ patients proposed for surgery, a total healing rate of 91.66% was obtained after the first surgery, only one "failure" was reported. | proposed association of methods, with superior clinical results compared to classical therapies. |

| | | | | |
|---|---|---|---|---|
| | | applied 3 consecutive days before surgery. The necrotic bone was removed using an ultrasonic device. PRF membranes were obtained for every patient prior to surgery. Patients were scheduled for periodical follow-up, for at least 6 months after the treatment. | | |
| Longo et al., 2014 [32] | A retrospective observational clinical study, with a follow-up range from 6 to 94 months, to evaluate PRP's therapeutic effect in promoting ONJ wounds healing. | 72 patients affected by ONJ, with every grade (0, 1, 2, or 3) of lesions underwent a two-week nonsurgical treatment. After the treatment, If the lesion had healed, a regular follow-up was performed, otherwise if the lesion had improved, they continued therapy for other two weeks; if the lesion had not improved or even worsened, they underwent surgical treatment (15 patients) or surgical treatment with PRP (34 patients), continuing the nonsurgical treatment. All the 72 patients thus underwent nonsurgical treatment. | For a stage 0 BRONJ, nonsurgical management was successful in every case (100%). Nonsurgical management success rate decreases in subsequent stages. As regards patients who underwent surgical procedures, PRP group was found statistically significantly more successful than the surgery without PRP group. | Good results shown by PRP in improving wound healing give a way to case-control randomized studies that could give definitive evidence of its effectiveness. |

Abbreviations: BFs, bisphosphonates; PRP, platelet rich plasma; ONC, oncologic; OST, osteometabolic; MRONJ, medication-related osteonecrosis of the jaw; PRF, platelet rich fibrin; L-PRF, leukocyte and platelet rich fibrin; ONJ. Osteonecrosis of the jaw; PMBT, photobiomodulation therapy; aPDT, antimicrobial photodynamic therapy; SPO, sequestrectomy with peripheral ostectomy; BRONJ, bisphosphonates-related osteonecrosis of the jaw; PRGF, plasma richi in growth factors; BTP, bisphosphonate therapy ; BMP-2, bone morphogenetic protein-2.

**Table S6.** NHLBI Quality Assessment of Controlled Intervention Studies.

| NHLBI Quality Assessment of Controlled Intervention Studies | | | | | | | | | | | | | | | | |
|---|---|---|---|---|---|---|---|---|---|---|---|---|---|---|---|---|
| **First Author et al., Year** | **Q1** | **Q2** | **Q3** | **Q4** | **Q5** | **Q6** | **Q7** | **Q8** | **Q9** | **Q10** | **Q11** | **Q12** | **Q13** | **Q14** | **Total Score** | **Quality Rating** |
| Mauceri et al., 2020 [14] | N | N | N | N | N | Y | Y | Y | Y | Y | Y | N | Y | Y | 8/14 (57,14%) | Fair |
| Asaka et al., 2017 [15] | N | N | N | N | N | Y | Y | Y | Y | Y | Y | Y | Y | Y | 9/14 (64,29%) | Fair |
| Parise et al., 2022 [16] | Y | Y | Y | N | N | Y | Y | Y | Y | Y | Y | N | Y | Y | 11/14 (78,57%) | Good |
| Miranda et al., 2021 [17] | N | N | N | N | N | Y | Y | Y | Y | Y | Y | N | Y | Y | 8/14 (57,14%) | Fair |
| Giudice et al., 2018 [18] | Y | Y | Y | N | N | Y | Y | Y | Y | Y | Y | N | Y | Y | 11/14 (78,557%) | Good |
| Tenore et al., 2020 [19] | N | N | N | N | N | Y | Y | Y | Y | Y | Y | N | Y | Y | 8/14 (57,14%) | Fair |
| Vescovi et al., 2015 [20] | N | N | N | N | N | Y | Y | Y | Y | Y | Y | N | Y | Y | 8/14 (57,14%) | Fair |
| Park et al., 2017 [21] | Y | Y | Y | N | N | Y | Y | Y | Y | Y | Y | N | Y | Y | 11/14 (78,557%) | Good |

Q1: Was the study described as randomized, a randomized trial, a randomized clinical trial, or an RCT?, Q2: Was the method of randomization adequate (i.e., use of randomly generated assignment)?, Q3: Was the treatment allocation concealed (so that assignments could not be predicted)?, Q4: Were study participants and providers blinded to treatment group assignment?, Q5: Were the people assessing the outcomes blinded to the participants' group assignments?, Q6: Were the groups similar at baseline on important characteristics that could affect outcomes (e.g., demographics, risk factors, co-morbid conditions)?, Q7: Was the overall drop-out rate from the study at endpoint 20% or lower of the number allocated to treatment?, Q8: Was the differential drop-out rate (between treatment groups) at endpoint 15 percentage points or lower?, Q9: Was there high adherence to the intervention protocols for each treatment group?, Q10: Were other interventions avoided or similar in the groups (e.g., similar background treatments)?, Q11: Were outcomes assessed using valid and reliable measures, implemented consistently across all study participants?, Q12: Did the authors report that the sample size was sufficiently large to be able to detect a difference in the main outcome between groups with at least 80% power?, Q13: Were outcomes reported or subgroups analyzed prespecified (i.e., identified before analyses were conducted)?, Q14: Were all randomized participants analyzed in the group to which they were originally assigned, i.e., did they use an intention-to-treat analysis?; Total Score: Number of yes; CD: cannot be determined; NA: not applicable; NR: not reported; N: no; Y: yes. Quality Rating: Poor <50%, Fair 50–75%, Good ≥75%.

**Table S7.** NHLBI Quality Assessment for Before-After (Pre-Post) Studies with No Control Group.

| NHLBI Quality Assessment Tool for Before-After (Pre-Post) Studies with No Control Group | | | | | | | | | | | | | | |
|---|---|---|---|---|---|---|---|---|---|---|---|---|---|---|
| **First Author et al., Year** | **Q1** | **Q2** | **Q3** | **Q4** | **Q5** | **Q6** | **Q7** | **Q8** | **Q9** | **Q10** | **Q11** | **Q12** | **Total Score** | **Quality Rating** |
| Sahin et al., 2020 [22] | Y | Y | Y | N | N | Y | Y | N | Y | Y | N | N | 7/12 (58,33%) | Fair |
| Merigo et al., 2018 [23] | Y | N | Y | Y | N | Y | Y | N | Y | Y | N | Y | 8/12 (66,67%) | Fair |

Q1: Was the study question or objective clearly stated?, Q2: Were eligibility/selection criteria for the study population prespecified and clearly described?, Q3: Were the participants in the study representative of those who would be eligible for the test/service/intervention in the general or clinical population of interest?, Q4: Were all eligible participants that met the prespecified entry criteria enrolled?, Q5: Was the sample size sufficiently large to provide confidence in the findings?, Q6: Was the test/service/intervention clearly described and delivered consistently across the study population?, Q7: Were the outcome measures prespecified, clearly defined, valid, reliable, and assessed consistently across all study participants?, Q8: Were the people assessing the outcomes blinded to the participants' exposures/interventions?, Q9: Was the loss to follow-up after baseline 20% or less? Were those lost to follow-up accounted for in the analysis?, Q10: Did the statistical methods examine changes in outcome measures from before to after the intervention? Were statistical tests done that provided p values for the pre-to-post changes?, Q11: Were outcome measures of interest taken multiple times before the intervention and multiple times after the intervention (i.e., did they use an interrupted time-series design)?, Q12: If the intervention was conducted at a group level (e.g., a whole hospital, a community, etc.) did the statistical analysis take into account the use of individual-level data to determine effects at the group level?; Total Score: Number of yes; CD: cannot be determined; NA: not applicable; NR: not reported; N: no; Y: yes. Quality Rating: Poor <50%, Fair 50–75%, Good ≥75%.

**Table S8.** NHLBI Quality Assessment Tool for Observational Cohort and Cross-Sectional Studies.

| | NHLBI Quality Assessment Tool for Observational Cohort and Cross-Sectional Studies | | | | | | | | | | | | | | |
|---|---|---|---|---|---|---|---|---|---|---|---|---|---|---|---|
| **First Author et al., Year** | **Q1** | **Q2** | **Q3** | **Q4** | **Q5** | **Q6** | **Q7** | **Q8** | **Q9** | **Q10** | **Q11** | **Q12** | **Q13** | **Q14** | **Total Score** | **Quality Rating** |
| Tartaroti et al., 2020 [24] | Y | Y | Y | N | Y | Y | Y | Y | Y | N | Y | N | Y | N | 11/14 (78.57%) | Good |
| Ozalp et al., 2021 [25] | Y | Y | Y | Y | Y | Y | Y | Y | Y | N | Y | N | Y | N | 11/14 (78,57%) | Good |
| Martins et al., 2012 [26] | Y | Y | Y | Y | Y | N | Y | Y | Y | N | Y | N | Y | N | 10/14 (71,42%) | Fair |
| Valente et al., 2019 [27] | Y | Y | Y | Y | Y | Y | Y | N | Y | N | Y | N | Y | Y | 11/14 (78.57%) | Good |
| Mauceri et al., 2018 [28] | Y | Y | Y | Y | Y | Y | Y | Y | Y | Y | Y | N | Y | N | 12/14 (85,7%) | Good |
| Sahin et al., 2021 [29] | Y | Y | Y | N | Y | N | N | N | Y | N | Y | N | Y | N | 7/14 (50%) | Fair |
| Vescovi et al., 2012 [30] | Y | Y | Y | Y | Y | Y | Y | N | Y | N | Y | N | Y | N | 10/14 (71,42%) | Fair |
| Nica et al., 2021 [31] | Y | Y | Y | N | Y | Y | Y | Y | Y | N | Y | N | Y | N | 10/14 (71,42%) | Fair |
| Longo et al., 2014 [32] | Y | Y | Y | Y | Y | Y | Y | Y | Y | N | Y | N | Y | N | 12/14 (85,71%) | Good |

Q1: Was the research question or objective in this paper clearly stated?, Q2: Was the study population clearly specified and defined?, Q3: Was the participation rate of eligible persons at least 50%?, Q4: Were all the subjects selected or recruited from the same or similar populations (including the same time period)? Were inclusion and exclusion criteria for being in the study prespecified and applied uniformly to all participants?, Q5: Was a sample size justification, power description, or variance and effect estimates provided?, Q6: For the analyses in this paper, were the exposure(s) of interest measured prior to the outcome(s) being measured?, Q7: Was the timeframe sufficient so that one could reasonably expect to see an association between exposure and outcome if it existed?, Q8: For exposures that can vary in amount or level, did the study examine different levels of the exposure as related to the outcome (e.g., categories of exposure, or exposure measured as continuous variable)?, Q9: Were the exposure measures (independent variables) clearly defined, valid, reliable, and implemented consistently across all study participants?, Q10: Was the exposure(s) assessed more than once over time?, Q11: Were the outcome measures (dependent variables) clearly defined, valid, reliable, and implemented consistently across all study participants?, Q12: Were the outcome assessors blinded to the exposure status of participants?, Q13: Was loss to follow-up after baseline 20% or less?, Q14: Were key potential confounding variables measured and adjusted statistically for their impact on the relationship between exposure(s) and outcome(s)?; Total Score: Number of yes; CD: cannot be determined; NA: not applicable; NR: not reported; N: no; Y: yes. Quality Rating: Poor <50%, Fair 50–75%, Good ≥75%.

**References**


1. Reis C.H.B., Buchaim D.V., Ortiz A.C., Fideles S.O.M., Dias J.A, Miglino M.A., Teixeira D.B., Pereira E.S.B.M., da Cunha M.R., Buchaim R.L. Application of Fibrin Associated with Photobiomodulation as a Promising Strategy to Improve Regeneration in Tissue Engineering: A Systematic Review. *Polymers (Basel).* **2022**, 14, 3150.
2. Razavi P., Jafari A., Vescovi P., Fekrazad R. Efficacy of Adjunctive Photobiomodulation in the Management of Medication-Related Osteonecrosis of the Jaw: A Systematic Review. *Photobiomodul Photomed Laser Surg*. **2022**, 40, 777-791.
3. Cano-Durán J.A., Peña-Cardelles J.F., Ortega-Concepción D., Paredes-Rodríguez V.M., García-Riart M., López-Quiles J. The role of Leucocyte-rich and platelet-rich fibrin (L-PRF) in the treatment of the medication-related osteonecrosis of the jaws (MRONJ). *J Clin Exp Dent.* **2017**, 9, e1051-e1059.
4. De Santis D., Gelpi F., Luciano U., Zarantonello M., Poscolere A., Modena N., Faccioni P., Causarano G., Finotti M., Zotti F., Magi M., Iurlaro A., Nocini P.F., Alberti C., Zangani A., Capocasale G., Donadello D., Nocini R., Bernardello F. New trends in adjunctive treatment and diagnosis in medication-related osteonecrosis of the jaw: A 10-year review. *J Biol Regul Homeost Agents*. **2020**, 34, 37-48.
5. Mijiritsky E., Assaf H.D., Kolerman R., Mangani L., Ivanova V., Zlatev S. Autologous Platelet Concentrates (APCs) for Hard Tissue Regeneration in Oral Implantology, Sinus Floor Elevation, Peri-Implantitis, Socket Preservation, and Medication-Related Osteonecrosis of the Jaw (MRONJ): A Literature Review. *Biology (Basel)*. **2022**, 11, 1254.
6. Rusilas H., Balčiūnaitė A., Žilinskas J. Autologous platelet concentrates in treatment of medication related osteonecrosis of the jaw. *Stomatologija.* **2020**, 22, 23-27.
7. Lopez-Jornet P., Sanchez Perez A., Amaral Mendes R., Tobias A. Medication-related osteonecrosis of the jaw: Is autologous platelet concentrate application effective for prevention and treatment? A systematic review. *J Craniomaxillofac Surg*. **2016**, 44, 1067-72.
8. Fortunato L., Bennardo F., Buffone C., Giudice A. Is the application of platelet concentrates effective in the prevention and treatment of medication-related osteonecrosis of the jaw? A systematic review. *J Craniomaxillofac Surg*. **2020**, 48, 268-285.
9. Hao L., Tian Z., Li S., Yan K., Xue Y. Osteonecrosis of the jaw induced by bisphosphonates therapy in bone metastases patient: Case report and literature review. *Oral Oncol*. **2022**, 128, 105852.
10. de Souza Tolentino E., de Castro T.F., Michellon F.C., Passoni A.C.C., Ortega L.J.A., Iwaki L.C.V., da Silva M.C. Adjuvant therapies in the management of medication-related osteonecrosis of the jaws: Systematic review. *Head Neck*. **2019**, 41, 4209-4228.
11. Goker F., Grecchi E., Grecchi F., Francetti L., Del Fabbro M. Treatment of medication-related osteonecrosis of the jaw (MRONJ). A systematic review. *Eur Rev Med Pharmacol Sci.* **2021**, 25, 2662-2673.
12. Momesso G.A.C., Lemos C.A.A., Santiago-Júnior J.F., Faverani L.P., Pellizzer E.P. Laser surgery in management of medication-related osteonecrosis of the jaws: a meta-analysis. *Oral Maxillofac Surg*. **2020,** 24, 133-144
13. Li F.L., Wu C.B., Sun H.J., Zhou Q. Effectiveness of laser-assisted treatments for medication-related osteonecrosis of the jaw: a systematic review. *Br J Oral Maxillofac Surg.* **2020**, 58, 256-267.

14. Mauceri, R.; Panzarella, V.; Pizzo, G.; Oteri, G.; Cervino, G.; Mazzola, G.; Di Fede, O.; Campisi, G. Platelet-Rich Plasma (PRP) in dental extraction of patients at risk of bisphosphonate-related osteonecrosis of the jaws: A two-year longitudinal study. *Appl. Sci.***2020**, *10*, 4487.

15. Asaka T., Ohga N., Yamazaki Y., Sato J., Satoh C., Kitagawa Y. Platelet-rich fibrin may reduce the risk of delayed recovery in tooth-extracted patients undergoing oral bisphosphonate therapy: a trial study. *Clin Oral Investig*. **2017,** 21, 2165-2172.
16. Parise G.K., Costa B.N., Nogueira M.L., Sassi L.M., Schussel JL. Efficacy of fibrin-rich platelets and leukocytes (L-PRF) in tissue repair in surgical oral procedures in patients using zoledronic acid-case-control study. *Oral Maxillofac Surg*. **2022**, 24, 1–6.
17. Miranda M., Gianfreda F., Raffone C., Antonacci D., Pistilli V., Bollero P. The Role of Platelet-Rich Fibrin (PRF) in the Prevention of Medication-Related Osteonecrosis of the Jaw (MRONJ). *Biomed Res Int*. **2021**, 4948139.
18. Giudice A., Barone S., Giudice C., Bennardo F., Fortunato L. Can platelet-rich fibrin improve healing after surgical treatment of medication-related osteonecrosis of the jaw? A pilot study. *Oral Surg Oral Med Oral Pathol Oral Radiol.* **2018**, 126, 390-403.
19. Tenore G., Zimbalatti A., Rocchetti F., Graniero F., Gaglioti D., Mohsen A., Caputo M., Lollobrigida M., Lamazza L., De Biase A., Barbato E., Romeo U. Management of Medication-Related Osteonecrosis of the Jaw (MRONJ) Using Leukocyte- and Platelet-Rich Fibrin (L-PRF) and Photobiomodulation: A Retrospective Study. *J Clin Med*. **2020**, 9, 3505.
20. Vescovi P., Meleti M., Merigo E., Manfredi M., Fornaini C. Tooth Extractions in High-Risk Patients Under Bisphosphonate Therapy and Previously Affected With Osteonecrosis of the Jaws: Surgical Protocol Supported by Low-Level Laser Therapy. *J Craniofac Surg*. **2015,** 26, 696–699.
21. Park J.H., Kim J.W., Kim S.J. Does the Addition of Bone Morphogenetic Protein 2 to Platelet-Rich Fibrin Improve Healing After Treatment for Medication-Related Osteonecrosis of the Jaw? *J Oral Maxillofac Surg.* **2017,** 75, 1176-1184.
22. Sahin, O.; Tatar, B.; Ekmekciog˘lu, C.; Aliyev, T.; Odabas ̧i, O. Prevention of medication related osteonecrosis of the jaw afterdentoalveolar surgery: An institution's experience. *J. Clin. Exp. Dent.* **2020**, *12*, e771–e776.
23. Merigo E., Cella L., Oppici A., Cristina Arbasi M., Clini F., Fontana M., Fornaini C. Combined Approach to Treat Medication-Related Osteonecrosis of the Jaws. *J Lasers Med Sci*. **2018**, 9, 92-100.
24. Tartaroti N.C., Marques M.M., Naclério-Homem M.D.G., Migliorati C.A., Zindel Deboni M.C. Antimicrobial photodynamic and photobiomodulation adjuvant therapies for prevention and treatment of medication-related osteonecrosis of the jaws: Case series and long-term follow-up. *Photodiagnosis Photodyn Ther.* **2020,** 29, 101651.
25. Özalp Ö., Yıldırımyan N., Öztürk C., Kocabalkan B., Şimşek Kaya G., Sindel A., Altay M.A. Promising results of surgical management of advanced medication related osteonecrosis of the jaws using adjunctive leukocyte and platelet rich fibrin. *BMC Oral Health*. **2021**, 21, 613.
26. Martins M.A., Martins M.D., Lascala C.A., Curi M.M., Migliorati C.A., Tenis C.A., Marques M.M. Association of laser phototherapy with PRP improves healing of bisphosphonate-related osteonecrosis of the jaws in cancer patients: a preliminary study. *Oral Oncol.* **2012,** 48, 79-84.
27. Valente N.A., Chatelain S., Alfonsi F., Mortellaro C., Barone A. Medication-Related Osteonecrosis of the Jaw: The Use of Leukocyte-Platelet-Rich Fibrin as an Adjunct in the Treatment. *J Craniofac Surg*. **2019**, 30, 1095-1101.
28. Mauceri R., Panzarella V., Maniscalco L., Bedogni A., Licata M.E., Albanese A., Toia F., Cumbo E.M.G., Mazzola G., Di Fede O., Campisi G. Conservative Surgical Treatment of Bisphosphonate-Related Osteonecrosis of the Jaw with Er,Cr:YSGG Laser and Platelet-Rich Plasma: A Longitudinal Study. *Biomed Res Int.* **2018**, 3982540.
29. Şahin O., Akan E., Tatar B., Ekmekcioğlu C., Ünal N., Odabaşı O. Combined approach to treatment of advanced stages of medication-related osteonecrosis of the jaw patients. *Braz J Otorhinolaryngol.* **2022**, 88, 613-620.

30. Vescovi P., Manfredi M., Merigo E., Guidotti R., Meleti M., Pedrazzi G., Fornaini C., Bonanini M., Ferri T., Nammour S. Early surgical laser-assisted management of bisphosphonate-related osteonecrosis of the jaws (BRONJ): a retrospective analysis of 101 treated sites with long-term follow-up. *Photomed Laser Surg.* **2012**, 30, 5-13.
31. Nica D.F., Riviș M., Roi C.I., Todea C.D., Duma V.F., Sinescu C. Complementarity of Photo-Biomodulation, Surgical Treatment, and Antibiotherapy for Medication-Related Osteonecrosis of the Jaws (MRONJ). *Medicina (Kaunas).* **2021,** 57, 145.
32. Longo F., Guida A., Aversa C., Pavone E., Di Costanzo G., Ramaglia L., Ionna F. Platelet rich plasma in the treatment of bisphosphonate-related osteonecrosis of the jaw: personal experience and review of the literature. *Int J Dent.* **2014**, 298945.
